# Rapid Diagnosis of Aneuploidy Using Segmental Duplication Quantitative Fluorescent PCR

**DOI:** 10.1371/journal.pone.0088932

**Published:** 2014-03-13

**Authors:** Xiangdong Kong, Lin Li, Lei Sun, Kepeng Fu, Ju Long, Xunjin Weng, Xuehe Ye, Xinxiong Liu, Bo Wang, Shanhuo Yan, Haiming Ye, Zuqian Fan

**Affiliations:** 1 Prenatal Diagnosis Center, the First Affiliated Hospital of Zhengzhou University, Henan, China; 2 Department of Genetic Laboratory, Lin Yi People’s Hospital, Shandong, China; 3 Laboratory of Medical Genetics, Qinzhou Maternal and Child Health Care Hospital, Guangxi, China; 4 Genetics Laboratory, Hubei Maternal and Child Health Hospital, Hubei, China; University of Bonn, Institut of experimental hematology and transfusion medicine, Germany

## Abstract

The aim of this study was use a simple and rapid procedure, called segmental duplication quantitative fluorescent polymerase chain reaction (SD-QF-PCR), for the prenatal diagnosis of fetal chromosomal aneuploidies. This method is based on the co-amplification of segmental duplications located on two different chromosomes using a single pair of fluorescent primers. The PCR products of different sizes were subsequently analyzed through capillary electrophoresis, and the aneuploidies were determined based on the relative dosage between the two chromosomes. Each primer set, containing five pairs of primers, was designed to simultaneously detect aneuploidies located on chromosomes 21, 18, 13, X and Y in a single reaction. We applied these two primer sets to DNA samples isolated from individuals with trisomy 21 (n = 36); trisomy 18 (n = 6); trisomy 13 (n = 4); 45, X (n = 5); 47, XXX (n = 3); 48, XXYY (n = 2); and unaffected controls (n = 40). We evaluated the performance of this method using the karyotyping results. A correct and unambiguous diagnosis with 100% sensitivity and 100% specificity, was achieved for clinical samples examined. Thus, the present study demonstrates that SD-QF-PCR is a robust, rapid and sensitive method for the diagnosis of common aneuploidies, and these analyses can be performed in less than 4 hours for a single sample, providing a competitive alternative for routine use.

## Introduction

Chromosomal aneuploidies cause a number of syndromes attributable to chromosomal aberration, and these abnormalities are typically associated with severe mental retardation, multiple dysmorphic features, growth retardation, etc. The frequency of chromosomal aneuploidy is approximately 1 out of 160 live births in the human population [1]. These diseases cannot be cured, and prenatal diagnosis is important to predict these syndromes. The prenatal diagnosis of chromosome aneuploidies is typically performed using G-band cytogenetic analysis with *in vitro* cultures of nucleated fetal cells retrieved through amniocentesis, chorionic biopsy or fetal blood sampling. This conventional cytogenetic technique detects a wide range of aberrations with high reliability and has become the gold standard for the detection of fetal chromosomal abnormalities since the 1970s [2]. However, conventional cytogenetics is time consuming (up to 2 weeks) and has technical issues, such as culture failure or external contamination. This method is also unable to detect submicroscopic duplications and deletions.

Quantitative methods, measuring the contributions from two different chromosomes, have been used for the rapid detection of trisomy 21 and other chromosome abnormalities. These methods target non-polymorphic sequences rather than polymorphisms, making this method potentially applicable to all human populations [3]. In 1997, homologous gene quantitative polymerase chain reaction (HGQ-PCR) was devised to estimate chromosome numbers for the detection of trisomy 21, showing unique advantages to detection using a single pair of primers [4]. However, a major drawback of this method is that the sequence of the sense primer differs from the PFKL-CH21 gene in exon 8 by one nucleotide, and the antisense primer differs from the PFKM-CH1 gene in exon 9 by one nucleotide. Therefore, the primers could not perfectly bind to the target sequence, making it difficult to maintain the original ratio. Real-time quantitative PCR (RT-PCR) is one of the most robust and versatile tools for the measurement of the copy numbers of nucleic acid sequences. This technology is amenable to automation and high throughput and could potentially reduce turnaround time and avoid issues of contamination [5]. Unfortunately, when dual primer sets and dual Taqman probes are used in a single reaction for amplification, even subtly biased annealing temperatures or DNA quality could produce false negative or ambiguous results [6]. Paralogous sequence quantification and high-resolution melting analyses are alternative techniques based on the use of a single primer to simultaneously amplify sequences from paralogous genes or segmental duplications [7], [8]. These tests maintain the original ratio between the target and control chromosomes and can clearly differentiate between samples from patients with common aneuploidies and those from unaffected controls. However, these two tests require several separate PCR reactions for each sample, reducing sample throughput and increasing the probability of handling errors.

Quantitative fluorescence polymerase chain reaction (QF-PCR) is highly accurate and robust, processing 96 samples at one time in an automated system, and the results are available in less than 48 hours [9], [10]. However, a major drawback to this technique is that the informative polymorphisms observed in one human population might not be applicable in another [11]–[13]. Multiplex ligation-dependent probe amplification (MLPA) has been validated for the diagnosis of changes in genomic copy numbers, and this technique has recently been applied for the analysis of chromosomal aneuploidy [14]. However, MLPA requires an overnight step for hybridization to human genomic DNA, making this technique time consuming and difficult to develop [15],[16].

To address these drawbacks, we developed two novel segmental duplications for each target chromosome (chromosomes 21, 18, 13, X and Y) to detect aneuploidies. Segmental duplications are two similar sequences with different fragment lengths, located on two different chromosomes. These sequences maintain the original ratio between the two different chromosomes when co-amplified using a single pair of fluorescent primers. The PCR products of different sizes are subsequently analyzed through capillary electrophoresis, and the aneuploidies are determined based on the relative dosage between the two chromosomes in a single reaction. In the present study, we applied this method, called segmental duplication quantitative fluorescent polymerase chain reaction (SD-QF-PCR), to analyze 96 DNA samples. Here, we show that this method is a reliable, simple and high-throughput alternative for the diagnosis of targeted aneuploidies.

## Materials and Methods

### Ethics Statement

This study was conducted according to the principles expressed in the Declaration of Helsinki. The protocol for this study was approved through the Research Ethics Committee of Qinzhou Maternal and Child Health Hospital. Written informed consent was obtained from each participant for the collection of samples and subsequent analyses.

### Samples

A total of 96 samples were collected in this study, of which 36 samples were obtained from patients with trisomy 21 (15 peripheral blood samples; 15 uncultured amniotic fluid samples and 6 cord blood samples); 2 samples were obtained from unbalanced translocation cases, including 46,XX,der(14;21)(q10; q10),+21; 4 cord blood samples were obtained from patients with trisomy 13; 6 samples from patients with trisomy 18 (3 uncultured amniotic fluid samples and 3 cord blood samples); 5 peripheral blood samples were obtained from 45, X patients; 3 peripheral blood samples were obtained from 47, XXX patients; and 2 peripheral blood samples were obtained from 48, XXYY patients. The samples obtained from 40 unaffected individuals (10 peripheral blood samples, 15 uncultured amniotic fluid samples and 15 cord blood samples) were used as unaffected controls. The karyotypes for all samples were previously validated through full karyotyping analysis, and these data were withheld from the technician who performed the SD-QF-PCR assays.

### DNA Extraction

DNA was extracted from uncultured amniotic fluid samples and blood samples using the QIAamp DNA Mini Kit (Qiagen) and the QIAamp DNA blood mini kit (Qiagen) according to the manufacturer’s instructions. Approximately 5–20 ng of DNA was diluted to 25–50 µL and used in a SD-QF-PCR multiplex-based reaction. The concentration of the extracted genomic DNA was spectrophotometrically determined at an absorbance of 260 nm (Quawell). A total of 96 DNA samples, stored at −80°C, were used in the QF-PCR analysis.

### Segmental Duplications and Primers

The segmental duplication sequences were obtained from two public databases: the Segmental Duplication Database (http://humanparalogy.gs.washington.edu/) and NCBI (http://www.ncbi.org/). Two independent primer sets (two segmental duplications per chromosome) were designed to simultaneously detect aneuploidies. The sequences of the PCR primers, available from the NCBI database, are presented in Table 1. One primer was unlabeled, and the other primer was labeled with FAM (6-carboxyfluorescein). All PCR primers and probes were constructed and purified through HPLC at GenScript.

**Table 1:** Information for primers and amplicons.

Segmental duplications	Amplicon location	Amplicon size (bp)	Primer sequence (5′→3′)
Primer set one 21/11	chr21∶38471113-38471259	147	F:GTGCCATTGACACAGGAGGAC
	chr11∶66961762-66961889	128	R:FAM-CTTTACCCCCAGCTGTCCC
Primer set two 21/6	chr21∶33921446-33921704	259	F:FAM-CAGGACCTGACCCTGGCT
	chr6∶156574766-156575012	247	R:CGGGAGACTGCCATTGATGA
Primer set one 18/10	chr18∶1362392-1362584	193	F:AGAGGACACACCAAACTAGATCA
	chr10∶89514395-89514555	161	R:FAM-AGATGAAATTCGGCCTGTTCA
Primer set two 18/1	chr18∶25655313-25655497	185	F:AGAGCTTCAGTTTCTCTGGTCC
	chr1∶60749715-60749904	190	R:FAM-TCTAGATCATTGCCCATTGCCC
Primer set one X/Y	chrX:11314897-11315111	215	F:TTGAGGCCAACCATCAGAGC
	chrY:6737876-6738096	221	R:FAM-AGCTACCACCTCATCCTGGG
Primer set two X/Y	chrX:90738412-90738622	211	F:TCTTTTCTACCACTGAAACAGAGT
	chrY:4592828-4593027	200	R:FAM-AGGCGAGTAGCTGTCTATGA
Primer set one 13/11	chr13∶90824978-90825215	238	F:AGGAATTCATCTTTCAAGGTCAAAA
	chr11∶13751134-13751377	244	R:FAM-TGTTTTGAAGCAGGAGGATTTCT
Primer set two 13/9	chr13∶19635510-19635629	120	F:CTTGGGAGAGGCCAAGAAAGA
	chr9∶96393435-96393536	102	R:FAM-TGAGGCTGGGTCGAGGG
Primer set one 3/X	chr3∶25797801-25798115	315	F:FAM-GGTTTTGCCTAGGTCCAGTG
	chrX:77386140-77386460	321	R:CCTGGTAATACAGCTCAGTGTCA
Primer set two 18/X	chr18∶30092561-30092811	251	F:GCACCAGTGAAGATGATGGC
	chrX:79816393-79816660	268	R:FAM-TTGACCTGCCATACGAAGCA

### PCR

The PCR amplification was performed using the Gene Amp® PCR System 9700 (Applied Biosystems) in a total reaction volume of 25 µL containing 1×Reaction Master Mix (Tiangen Biotech), 10 ng genomic DNA and 0.5 µmol/L of each forward and reverse primer. The reaction mixture was preheated at 95°C for 3 minutes, followed by 28 cycles of 30 seconds at 95°C, 30 seconds at 60°C, and 30 seconds at 72°C and a final extension step at 72°C for 10 minutes.

### Electrophoretic Analysis

**Figure 1:**
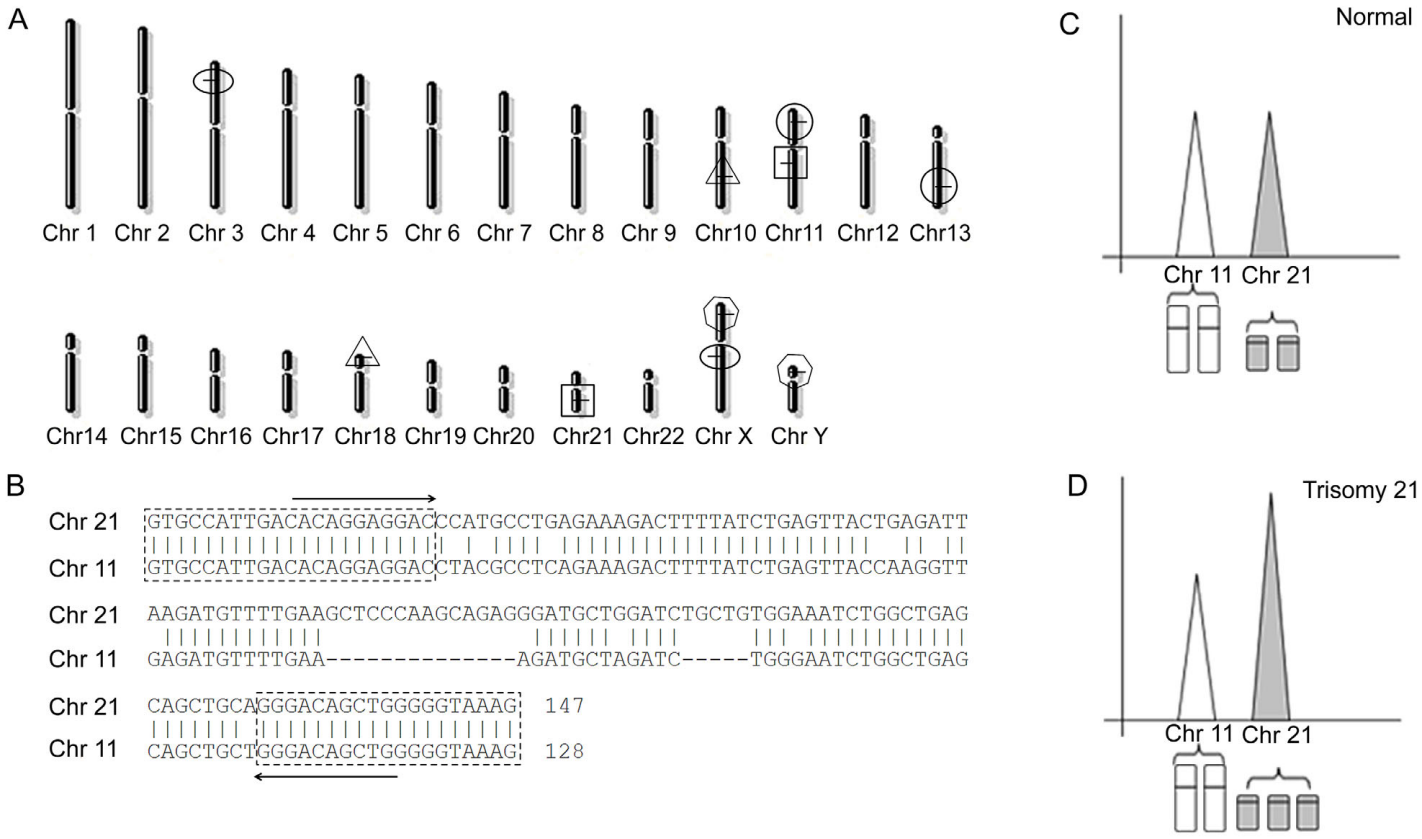
Ideogram of the segmental duplicates. A) The segmental duplications of primer set one located on human chromosomes (Chr). B) A single pair of primers was used to simultaneously amplify a 128-bp sequence on chr 11 and a 147-bp sequence on chr 21 from segmental duplications. C) Normal samples with a 1∶1 pattern ratio of chromosome dosages. D) Trisomy 21 samples with a 2∶3 pattern ratio of chromosome dosages.

Approximately 1 µL of the PCR product was mixed with 24 µL of formamide and 1 µL of the GeneScan 500 Rox size standard (Applied Biosystems). The mixture was denatured at 95°C for 3 minutes and placed on ice to prevent re-annealing until further analysis. The electrophoretic analysis was performed using a POP4 gel (Applied Biosystems) on the ABI 3130×l Genetic Analyzer (Applied Biosystems). The PCR products were separated and visualized using GeneScan Analysis software (Applied Biosystems). GeneMapper® ID Software v3.2 (Applied Biosystems) was used for the data analysis. The relative probe signal ratios were calculated based on the peak area of the segmental duplication (length). The expected value for a euploid sample is 1, and the expected value for a trisomic sample is 1.5, reflecting the additional target chromosome (Figure 1C).

## Results and Discussion

### Segmental Duplication Selection and Primer Design

Segmental duplications are nearly identical DNA segments widespread throughout the human genome [17],[18]. The information for segmental duplications was acquired from the Segmental Duplication Database (http://humanparalogy.gs.washington.edu/). We selected sequences with two hits from the whole genome: one hit must be located on the target chromosome (e.g., primer set one), and the other hit, with different sizes, must be located on a chromosome other than the target chromosome (Figure 1A). In addition, We built a consensus sequence, and blasted this sequence against the human genome in NCBI (http://www.ncbi.nlm.nih.gov/tools/primer-blast) to obtain a suitable pair of primers, matching both sequences (Figure 1B), which can maintain the original ratio between the target and control chromosomes during amplification (Figure 1C).

We originally designed more than ten pairs of primers for capillary electrophoresis, and these primers were pre-screened under a number of PCR conditions. The following criteria were used to select the primers: (a) no obvious nonspecific primer extension products when the segmental duplications were amplified; (b) segmental duplications were amplified with equal efficiency, with sequence quantifications resulting in a 1∶1 ratio; (c) a clear, non-overlapping difference between normal and abnormal samples after electrophoresis; and (d) minimal deviation from the mean. After PCR and capillary electrophoresis, we generated two primer sets (2 primers for each type of aneuploidy) with five pairs of primers each. Each primer met the criteria described above, and both primer sets could simultaneously detect the aneuploidies of chromosomes 21, 18, 13, X and Y in a single reaction (Table 1).

While we did not observe SNPs or CNVs within the target regions of the designed primers, these sequences might appear in some samples due to individual differences. Sequence variants in the primer region could produce false negative or ambiguous results. To resolve this problem, we analyzed two segmental duplications per chromosome to provide further accuracy in the analysis of a given aneuploidy. To reduce the mutual interference of the primers (which might result in imbalanced amplification and a failed experiment), we designed two independent primer sets to simultaneously detect aneuploidies. Thus, this assay design reduces the probability of detecting sequence variants in the two target regions at the same time. Furthermore, in clinical practice, differences between the two assays might reflect structural abnormalities. In such cases, full karyotyping, FISH, or array comparative genomic hybridization should be applied for diagnostic confirmation. The major drawback of this approach, similar to many other PCR-based assays, is that it is possible to miss some cases of structural abnormalities, low-level mosaicism or maternal cell contamination. However, this drawback does not hinder the wide application of this technology.

### Detection of the Common Chromosome Aneuploidies

**Figure 2:**
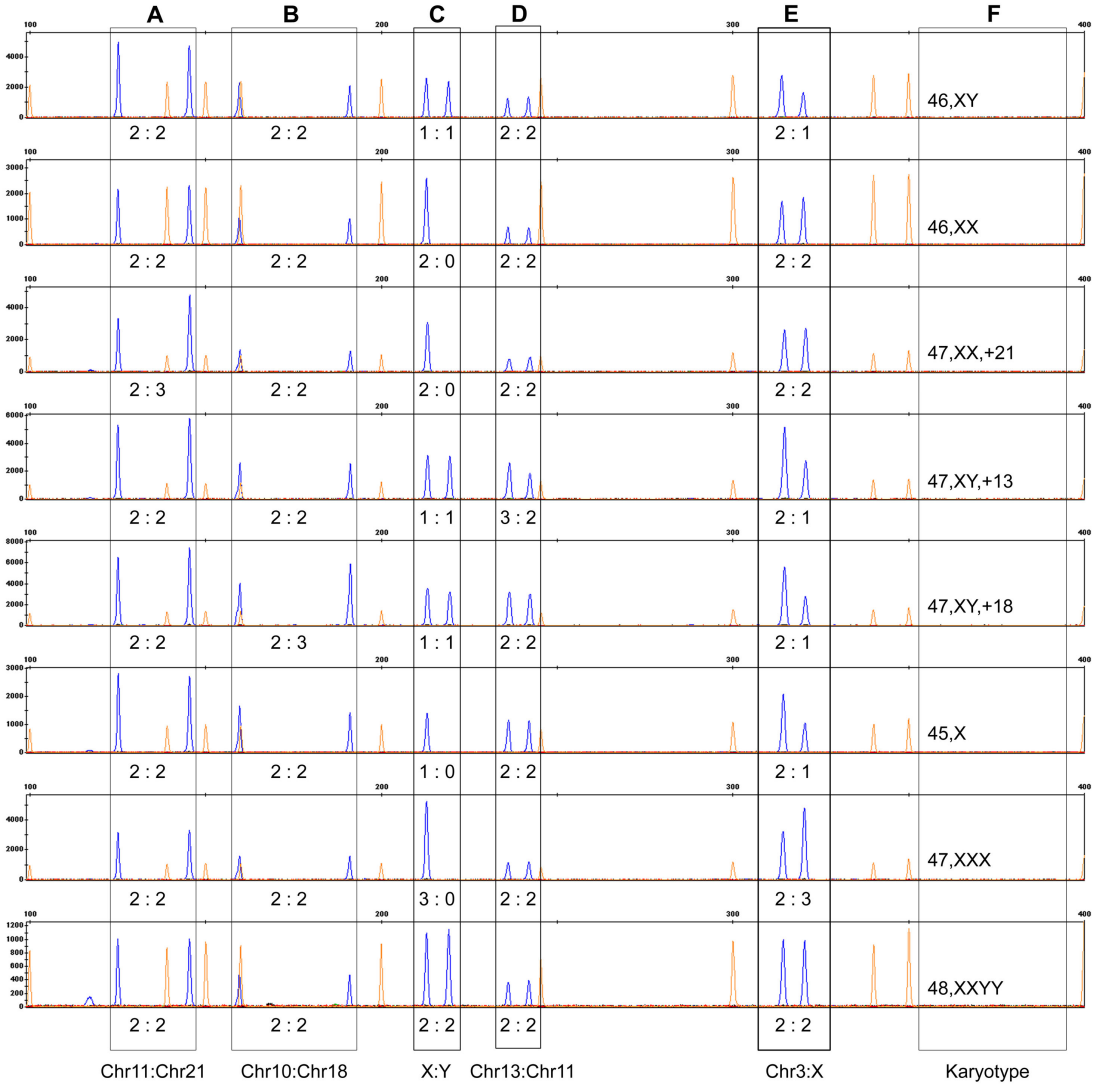
Electrophoretograms of primer set one. The aneuploidies of chromosomes 21, 18 and 13 could be detected using segmental duplications A, B and D, respectively; common sex chromosome aneuploidies could be identified through combinatorial analyses using segmental duplications C and E; F represents the karyotype of each individual.

To reduce the probability of a variant in the primer region, we selected two target regions per chromosome and designed two primer sets to simultaneously detect aneuploidies. For primer set one, a series of qualified segmental duplications, located on chromosomes 21 and 11, chromosomes 18 and 10 and chromosomes 13 and 11, were selected to detect the aneuploidies of chromosomes 21, 18 and 13, respectively (Figures 2A, 2B and 2D). To detect abnormalities on the sex chromosomes, we designed two pairs of primers: segmental duplications located on chromosomes X and Y (Figure 2C) were designed to quantify the ratio between the X and Y chromosomes, and segmental duplications located on chromosomes 3 and X were designed to quantify the ratio between chromosomes 3 and X (Figure 2E). In theory, the common abnormalities of sex chromosomes can be identified using these two primers pairs (Table 2).

**Table 2:** Assay patterns for common sex chromosome abnormalities.

Karyotype	Chr X:Chr Y	Autosome:Chr X
45,X	1∶0	2∶1
46,XX	2∶0	2∶2
46,XY	1∶1	2∶1
48,XXYY	2∶2	2∶2
47,XXX	3∶0	2∶3

**Figure 3:**
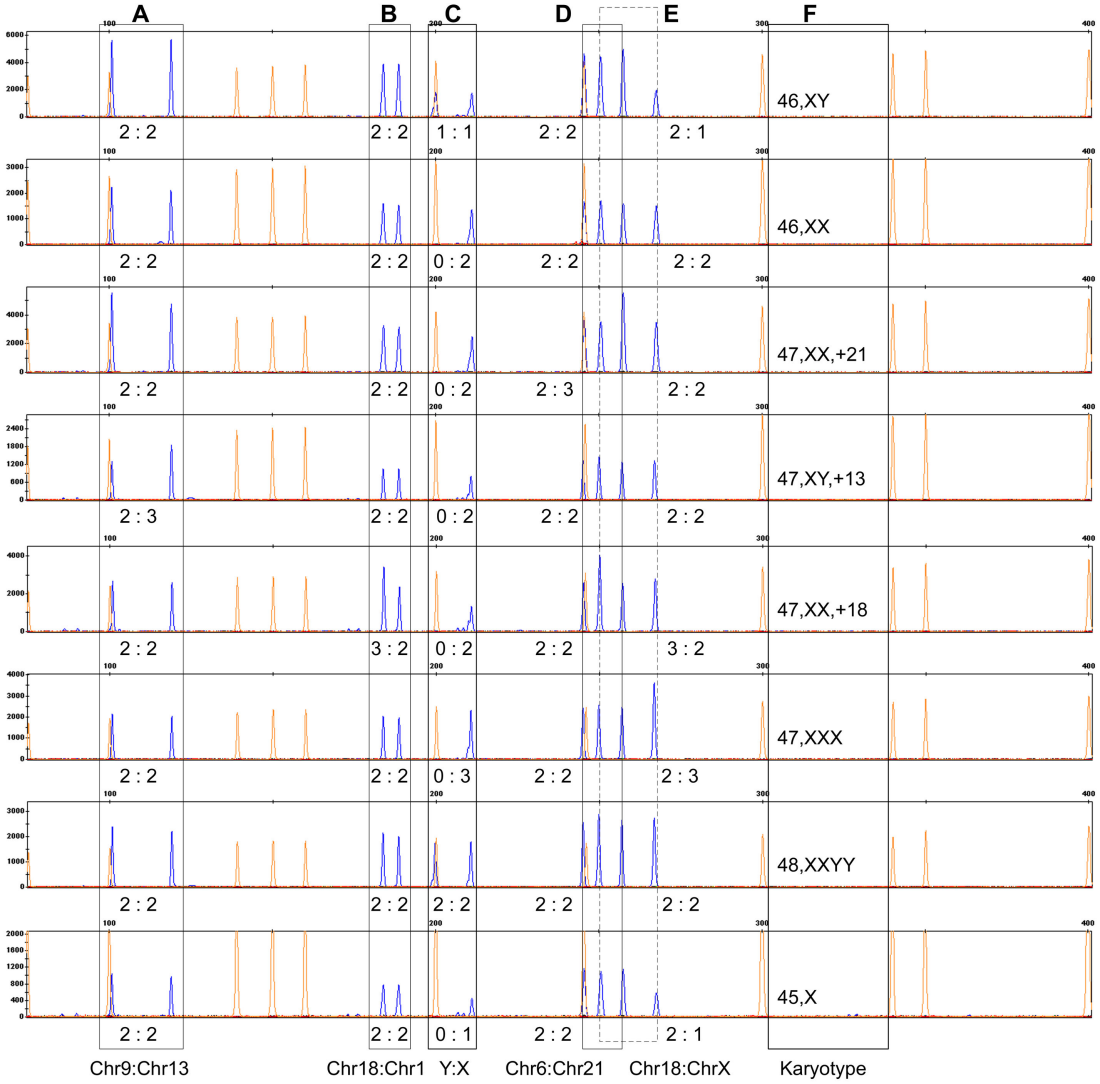
Electrophoretograms of primer set two. The aneuploidies of chromosomes 13, 18 and 21 could be detected using segmental duplications A, B and D, respectively; common sex chromosome aneuploidies could be identified through combinatorial analyses using segmental duplications C and E; F represents the karyotype of each individual.

For primer set two, the segmental duplications located on chromosomes 13 and 9, chromosomes 18 and 1 and chromosomes 21 and 6 were selected to detect the aneuploidies of chromosomes 13, 18 and 21, respectively (Figures 3A, 3B and 3D). Segmental duplications located on chromosomes X and 18 and chromosomes X and Y were designed to detect abnormalities on the sex chromosomes (Figures 3C and 3E).

**Figure 4:**
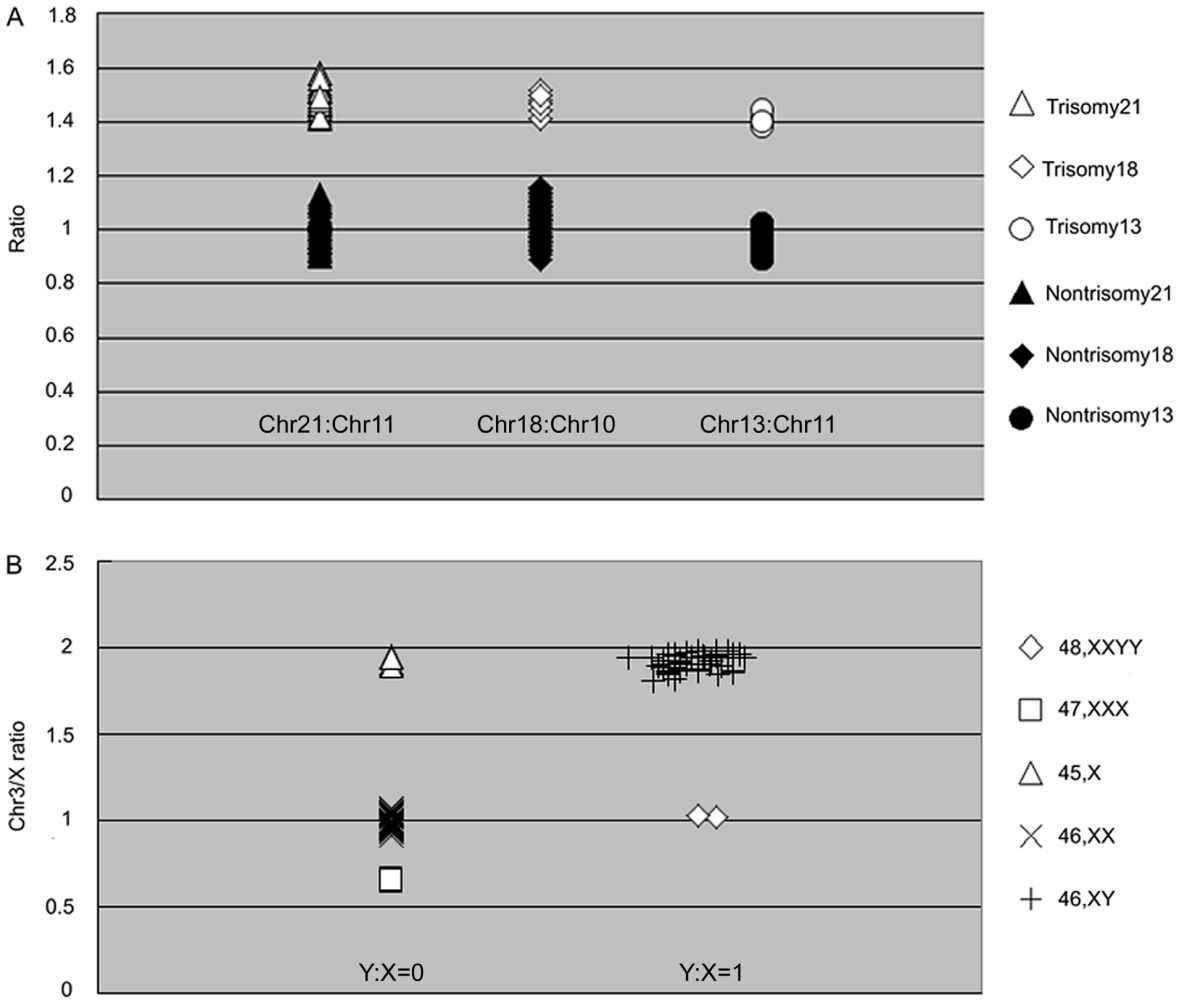
The individual distributions observed in the assays. A) Individual relative ratios of chromosomes 21,18 and 13 in normal and trisomic samples. B) Distribution of the X vs. Y and Chr 3 vs. X ratio; the x-axis represents the X/Y ratio, and the y-axis represents the Chr3/X ratio.

We examined 96 DNA samples in each of the two assays and obtained a correct and unambiguous diagnosis in all cases. In total, 56 aneuploidies were detected: 36 cases of trisomy 21; 4 cases of trisomy 13; 6 cases of trisomy 18; 5 cases of trisomy 45, X; 3 cases of trisomy 47, XXX; and 2 cases of trisomy 48, XXYY. A total of 40 unaffected DNA controls were also screened in each assay. The diagnostic sensitivity and specificity of all samples was 100%, and the results correlated with their respective karyotypes. We attribute this high reliably to the use of segmental duplications as detection targets. A single pair of primers can perfectly match both loci to co-amplify the segmental duplications located on two different chromosomes, while maintaining almost identical amplification efficiencies between target chromosomes and control chromosomes. Therefore, segmental duplications can be used to reliably detect chromosome abnormalities. For primer set one, the means and standard deviations for the segmental duplications on the autosomal chromosomes are shown in Table 3, and all 96 samples were categorized into different groups according to their mean values (Figure 4).

**Table 3:** Specificity and sensitivity of the autosomal chromosomes for primer set one.

Primer set	Primer set one		
Segmental duplication	Chr 21	Chr 18	Chr 13
Mean control	1.01	1.03	0.95
SD control	0.06	0.06	0.05
95% CI control	0.96–1.07	0.96–1.09	0.91–0.98
Mean trisomic	1.49	1.47	1.40
SD trisomic	0.05	0.04	0.03
95% CI trisomic	1.44–1.54	1.43–1.51	1.38–1.43
Total samples	96	96	96
Sensitivity	1	1	1
Specificity	1	1	1

A total of 36 samples with trisomy 21 were observed, including two unbalanced translocations (46,XX,der(14; 21)(q10;q10),+21) correctly diagnosed as trisomy 21 using SD-QF-PCR. For the determination of trisomy 21, two pairs of segmental duplications (Chr21/Chr11 and Chr21/Chr9) were selected for the identification of additional copies of chromosome 21 (Figure 2A and Figure 3D). The segmental duplications were located on the long arm of chromosome 21 [19]. Thus, these results demonstrate that this approach is not only an acceptable method for detecting an extra target chromosome, but also for detecting the relative dosage of the chromosomes in which the segmental duplications are located, i.e., chromosome deletion or duplication.

### Methodology Comparison

Fluorescent in situ hybridization (FISH) is a routine method used for the rapid detection of prenatal aneuploidies in interphase amniocytes. This method avoids the need to culture cells and reduces the time required for diagnosis. However, this technique is relatively labor intensive and requires technical expertise, requirements that prevent the efficient processing of a large number of samples in clinical diagnostic settings [20],[21]. These drawbacks have encouraged the development of more rapid and efficient methods for the detection of fetal chromosomal aneuploidies.

QF-PCR and MLPA are the most extensively validated DNA-based techniques described for the rapid detection of fetal chromosomal aneuploidies [9]–[12]. However, for QF-PCR, based on the amplification of polymorphic microsatellite repeats, the differences in the number of informative polymorphisms might limit the universality of this assay. Segmental duplications are not dependent on multiple informative polymorphism markers, applicable to all human populations. Thus, this method could be considered as an alternative or complement strategy to QF-PCR. MLPA, which requires an overnight step for hybridization, requires approximately 16 hours to detect a single sample. SD-QF-PCR exhibits equal application efficiency and saves more than 12 hours turnaround time, making SD-QF-PCR faster and easier. Compared with conventional karyotype analyses, SD-QF-PCR reduces the turnaround time from 2–4 weeks to less than 4 hours for a single aneuploidy test [2]. Using the current protocol, a single operator can manage at least 96 samples a day and report the results in less than 24 hours, which should meet the needs of most diagnostic laboratories.

## Conclusions

In the present study, we designed two novel sets of segmental duplications and described a simple and rapid SD-QF-PCR method as an alternative approach to simultaneously detect the aneuploidies of chromosomes 21, 18, 13, X and Y in a single reaction. This method can process 96 samples at a time in an automated system for each primer set, and the results are available the same day. The retrospective study of 96 DNA samples was entirely consistent with the previous results of conventional cytogenetics. Thus, this procedure represents a competitive alternative diagnostic tool for use in prenatal screening.
